# A common set of developmental miRNAs are upregulated in *Nicotiana benthamiana *by diverse begomoviruses

**DOI:** 10.1186/1743-422X-8-143

**Published:** 2011-03-29

**Authors:** Imran Amin, Basavaprabhu L Patil, Rob W Briddon, Shahid Mansoor, Claude M Fauquet

**Affiliations:** 1 Agricultural Biotechnology Division, National Institute for Biotechnology and Genetic Engineering (NIBGE), P O Box 577, Jhang Road, Faisalabad, Pakistan, ILTAB, Donald; 2 Donald Danforth Plant Science Center, St. Louis, MO 63132, USA

## Abstract

**Background:**

Begomoviruses are single-stranded DNA viruses that cause economically important diseases of many crops throughout the world and induce symptoms in plants, including enations, leaf curling and stunting, that resemble developmental abnormalities. MicroRNAs (miRNAs) are small endogenous RNAs that are involved in a variety of activities, including plant development, signal transduction and protein degradation, as well as response to environmental stress, and pathogen invasion.

**Results:**

The present study was aimed at understanding the deregulation of miRNAs upon begomovirus infection. Four distinct begomoviruses *African cassava mosaic virus *(ACMV), *Cabbage leaf curl virus *(CbLCuV), *Tomato yellow leaf curl virus *(TYLCV) and *Cotton leaf curl Multan virus*/Cotton leaf curl betasatellite (CLCuV/CLCuMB), were used in this study. Ten developmental miRNA were studied. *N. benthamiana *plants were inoculated with begomoviruses and their miRNA profiles were analysed by northern blotting using specific miRNA probes. The levels of most developmental miRNA were increased in *N. benthamiana *by TYLCV, CLCuMV/CLCuMB and CbLCuV infection with a common pattern despite their diverse genomic components. However, the increased levels of individual miRNAs differed for distinct begomoviruses, reflecting differences in severity of symptom phenotypes. Some of these miRNA were also common to ACMV infection.

**Conclusions:**

Our results have shown a common pattern of miRNAs accumulation upon begomovirus infection. It was found that begomoviruses generally increase the accumulation of miRNA and thus result in the decreased translation of genes involved in the development of plants. Identification of common miRNAs that are deregulated upon begomovirus infection may provide novel targets for control strategies aimed at developing broad-spectrum resistance.

## Background

MicroRNAs (miRNAs) are endogenous, approx. 22 nt RNAs that can play important regulatory roles in animals and plants by targeting mRNA for cleavage or translational repression [1]. miRNA are the second most abundant class of RNA among short RNAs [2] that play a very important role in multicellular organisms and influence the output of many protein-coding genes. The first miRNAs were discovered during a study of nematode larval development. Two approximately 22 nt RNAs (the *lin*-*4 *and *let*-*7 *RNAs) control developmental timing by binding to their respective mRNA targets preventing their translation [3,4].

In mid-2002, four groups reported RNAs with miRNA characteristics among the tiny RNAs present in *Arabidopsis *[5-8]. An important difference between plant and animal miRNAs is that the regulatory targets of plant miRNAs can be predicted with a fair degree of confidence, simply by identifying mRNAs with near perfect complementarity [9].

The discovery of miRNAs in plants is still an ongoing process. Much focus has been directed toward miRNA identification in *Arabidopsis *and rice, but many species which are important economically or evolutionarily have yet to be examined. Initial cloning of small RNAs from *Arabidopsis *and rice has revealed that plants are extremely rich in endogenous small RNAs and that only a small portion of cloned small RNAs correspond to miRNAs [6,10]. The majority of endogenous small RNA species represent small interfering RNAs (siRNAs). The difference between miRNAs and siRNAs lies in their biogenesis. miRNAs originate from the processing of single-stranded precursors that form a hairpin structure, whereas siRNAs are generated from long double-stranded RNAs (dsRNAs) or single-stranded RNAs that form hairpin structures [6].

Plant miRNAs have a high degree of sequence complementarity to their target mRNAs and direct the slicing of the target mRNAs in the middle of the complementary regions [10,11]. This has been demonstrated by the detection of 3' cleavage products that have 5' ends that start in the middle of the complementary regions. This is probably mediated by AGO1 [12,13]. However, plant miRNAs also regulate gene expression by translational repression [14-17].

Geminiviruses are an important group of plant viruses with small circular, single-stranded (ss) DNA genomes that replicate in the nucleus of infected cells [18]. Viruses of the family *Geminiviridae *are divided into four genera based on insect vectors and genome organization [19]. Whitefly-transmitted geminiviruses are classified in the genus *Begomovirus *and constitute the largest genus that causes economically-important diseases throughout the warmer parts of the world [19-21]. Begomoviruses originating from the New World are invariably bipartite, with genomes consisting of two ssDNA components, known as DNA A and DNA B, of approximately equal size (~2.8 kb). Although a few bipartite begomoviruses are known in the Old World, the vast majority of begomoviruses are monopartite with a genome that is a homolog of the DNA A component of the bipartite viruses, and most of these interact with ssDNA satellites [22]. Some begomoviruses, such as Tomato yellow leaf curl virus (TYLCV), are monopartite while a DNA satellite resembling betasatellite was found associated with Tomato leaf curl virus (ToLCV) [23].

Plant virus infections can result in disease symptoms that may include chlorosis and/or necrosis, curling, stunting and altered plant stature and morphology, presumably caused by interference of the infection with developmental processes [24]. In recent years, it has been proven experimentally that short RNA (sRNA), and particularly miRNAs, play important roles in plant development and are implicated in host-pathogen interactions [25,26].

Recent studies in plants and animals suggest that viruses can suppress gene expression and use endogenous RNA-silencing pathways to regulate host gene expression, presumably to benefit virus replication [26-29]. However, the underlying mechanisms that control these activities remain unclear. Epstein-Barr virus and other DNA viruses encode miRNAs that directly down- or up-regulate host and/or viral mRNAs [28].

Several studies have demonstrated that viral suppressors of RNA silencing can interfere with miRNA-mediated regulation of host genes [30,31]. These studies showed that viral proteins interfere with miRNA pathways, although it is unclear whether it is part of the virus replication strategy or a side effect due to the overlap of the siRNA and miRNA pathways.

Transgenic expression in plants of the AC4 protein from ACMV, a suppressor of post-transcriptional gene silencing (PTGS)[32], was correlated with decreased accumulation of host miRNAs and increased development abnormalities in *Arabidopsis thaliana *[33]. Down- regulation of miRNA correlated with an up-regulation of target mRNA level. Another study showed that infection of *N. tabacum *by plant RNA viruses representative of the *Tobamoviridae, Potyviridae*, and *Potexviridae *families altered accumulation of certain miRNAs [34].

In the present study the accumulation of ten miRNAs were studied as a result of the infection of four viruses belonging to different types of *begomovirus*. *African cassava mosaic virus *(ACMV; representing a bipartite virus from the Old World), *Cabbage leaf curl virus *(CbLCuV: representing a bipartite virus from New World), Tomato yellow leaf curl virus (TYLCV; representing a monopartite virus) and *Cotton leaf curl Multan virus*/cotton leaf curl Multan betasatellite (CLCuMV/CLCuMB: representing a betasatellite requiring monopartite virus) were used in this study. The accumulation of miRNA as a result of the infection of each virus was then compared to assess the possible role of specific miRNAs in virus pathogenicity.

## Results

### Virus infection

**Figure 1:**
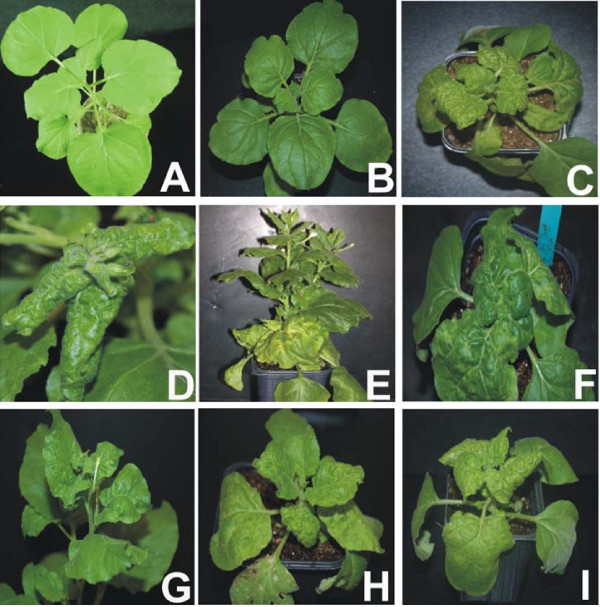
**Photographs of a healthy *N. benthamiana *plant (panel A), and *N. benthamiana *plants infected with PVX (panel B), CLCuMV/CLCuMB (panels C and D), ACMV (panels E and F) CbLCuV (panel G) and TYLCV (panels H and I)**. Photographs were taken at approx. 21 dpi.

*Potato virus X *(PVX) infection of *N. benthamiana *resulted in mild symptoms including mild vein yellowing, very mild vein thickening and a faint mosaic that appeared approx. 10 dpi. Additionally, at approx. 15 dpi, *N. benthamiana *plants infected with PVX ceased to show symptoms, indicative of recovery (Figure 1 panel B). Infection of CLCuMV/CLCuMB resulted in downward leaf curling, vein yellowing, stunting, vein swelling, and the formation of small enations on the veins on the undersides of leaves (Figure 1 panels C and D). *N. benthamiana *plants infected with CbLCuV showed mild symptoms after 16 dpi that include mild leaf curling and deformed leaves at the margins (Figure 1 panel G). TYLCV infection in *N. benthamiana *resulted in a stunted growth, severe downward leaf curling and vein yellowing (Figure 1 panels H and I). The presence of each virus was confirmed by PCR using specific primers designed to the replication-associated gene of each virus (data not shown).

### Effects of virus infection on miR156 levels

**Figure 2:**
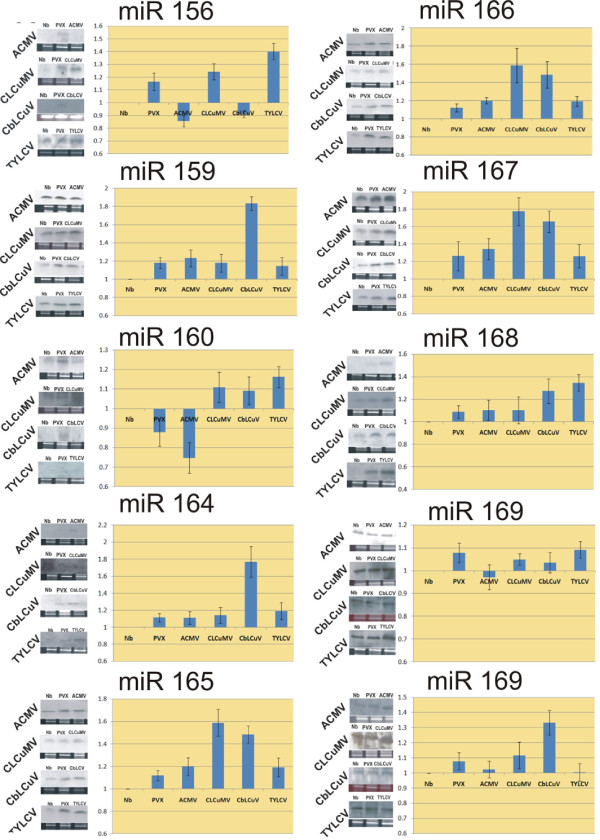
**Effects of virus infection on the levels of selected miRNAs**. Northern blot analysis to detect the accumulation of miR156, miR159, miR160, miR164, miR165, miR166, miR167, miR168, miR169 and miR170 after infection with begomoviruses and PVX. Shown below each blot is the rRNA band of the ethidium bromide stained agarose gel that was used to normalize the data for loading. The bar graphs show the average (with standard error) levels calculated for each miRNA levels for *N. benthamiana *plants infected with ACMV, CLCuMV/CLCuMB, CbLCuV, TYLCV and PVX. The relative levels of miRNA for virus infected plants were calculated by taking the levels in healthy *N. benthamiana *as 1.0. All samples were taken at 21 dpi.

Upon infection of *N. benthamiana *by ACMV and CbLCuV the level of miR156 decreases as compared to *N. benthamiana *while the infection of TYLCV and CLCuMV resulted in an increased accumulation of miR156 (Figure 2). PVX infection, which was used in this study as a control and a representative RNA virus, resulted in an increased accumulation of miR156 when compared with un-inoculated *N. benthamiana *plant (Figure 2).

#### - miR159

Infections of all begomoviruses under study resulted in an increase in the accumulation of miR159 when compared with healthy *N. benthamiana *plants. The highest increase was observed in case of the infection of CbLCuV (Figure 2). PVX infection also resulted in elevated level of miR159 as compared to healthy control (Figure 2).

#### - miR160

Infections of CbLCuV, TYLCV and CLCuMV resulted in a slight increase while ACMV resulted in decrease in the levels of miR160 when compared with healthy control plants (Figure 2). PVX infection also resulted in a slight decrease in the level of miR160 (Figure 2).

#### -miR164

*N. benthamiana *plants infected with ACMV, CLCuMV, CbLCuV and TYLCV showed an increase in the level of miR164 when compared with un-inoculated healthy plant. A maximum increase was observed in the infection of CbLCuV (Figure 2). Infection of PVX also resulted in an increase accumulation of miR164 (Figure 2).

#### -miR165 and miR166

Infection of *N. benthamiana *plants with ACMV, CLCuMV, CbLCuV and TYLCV resulted in an increase in the accumulation of miR165/166 when compared with the healthy control plants. The highest increase was observed in plants infected with CLCuMV (Figure 2). Infection of PVX also resulted in an increase in the accumulation of miR165/166 when compared with healthy *N. benthamiana *plants (Figure 2). However, this increase in accumulation of miR165/166 was less than for the begomovirus infection.

#### -miRNA 167

Infection with ACMV, CLCuMV, CbLCuV and TYLCV showed an increase in the accumulation of miR167 when compared with healthy control. Plants inoculated with PVX showed an increase in the level of miR167 though the increase was less than that caused by ACMV, CLCuMV and CbLCuV infections (Figure 2).

#### - miR168

Infection of *N. benthamiana *plants ACMV, CbLCuV, CLCuMV and TYLCV showed an increase in level of miR168 when compared with healthy plants. Infection of PVX also resulted in increased accumulation of miR167 which was comparable with ACMV and CLCuMV infections. A significant increase was observed for CbLCuV and TYLCV infections (Figure 2).

#### -miR 169 and miR170

**Figure 3:**
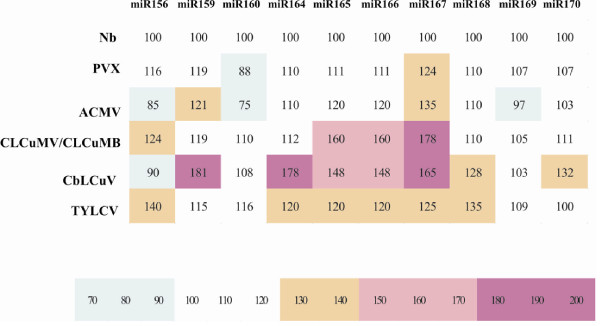
**A heat diagram summarizing the levels of miRNAs detected in *N. benthamiana *upon virus infection**. The signal of each band was quantified using imaj J and normalized with ethidium bromide stained RNA. The levels of miRNAs in *N. benthamiana *were taken as 100 and rest of the miRNA levels were calculated relative to that and color scheme is given which is also shown.

Infection of ACMV does not alter the level of miR169 significantly in *N. benthamiana *plants however, a slight decrease was observed when compared with healthy control plants (Figure 2). A slight increase in miR169 was observed in case of the infection of CLCuMV, CbLCuV and TYLCV (Figure 2). PVX infection in *N. benthamiana *also resulted in an increase in level of miR169 (Figure 2). A heat diagram summarizing the miRNA profiles in response to infection with the selected begomoviruses is given in Figure 3.

## Discussion

Recent studies of both animal and plant viruses have shown that viruses alter the RNA silencing pathway to regulate host gene expression [35,36]. One of the limitations at present is that the mechanisms controlling such activities are unclear. However, a generally accepted concept is that RNA silencing is a natural defense response of plants against invading viruses. To counter RNA silencing viruses encode certain proteins that can block the RNAi pathway and are referred to as suppressor of gene silencing [36,37]. It has been demonstrated that viral suppressors of RNA silencing can interact/interfere with the miRNA pathway [30,31], although it remains unclear whether these interactions are the part of the survival strategy of viruses or just side effects (collateral damage) of their infection cycle. In the work presented here the interaction begomoviruses ACMV, CLCuMV and its associated CLCuMB, TYLCV and CbLCuV with selected host miRNAs was studied. With the exception of CbLCuV, these viruses are well characterized as far as suppressors of gene silencing are concerned. TrAP and AC4 encoded by ACMV [32], the V2 protein encoded by TYLCV [38], the TrAP, C4 and V2 proteins of CLCuMV and βC1 encoded CLCuMB have been shown to have suppressor of gene silencing activity (Amin et al., manuscript in preparation).

miR156 has been shown to act on SQUAMOSA PROMOTER BINDING PROTEIN (SPL), which is believed to be a transcription factor [39,40]. The accumulation of miR156 upon infection by selected begomovirus showed that these viruses can be divided into two groups. Infections by the bipartite viruses (ACMV and CbLCuV) lead to a decrease in miR156 while infections by the monopartite viruses (CLCuMV and TYLCV) lead to an increase. It has been shown that SPL3 is a target of miR156 and constitutive expression of miR156 results in a prolonged juvenile vegetative phase and delayed flowering [40].

miR159 was identified independently by two groups [8,41] and is thought to target mRNAs coding for MYB proteins which are known to bind to the promoter of the floral meristem identity gene LEAFY (LFY; [9]). The LFY genes play an important role during the transition from the vegetative to the reproductive phase, as it is both necessary and sufficient for the initiation of individual flowers [42]. LFY is extensively expressed during the vegetative phase of plant growth [43]. Thus, the reduction in the expression of the LFY gene plays an important role in the transition from the vegetative phase to the sexual phase. A uniform pattern of up-regulation was observed for the accumulation of miR159 as a result of the infection of begomoviruses. These findings are in line with the recent findings where it was shown that upon infection of Tomato leaf curl New Delhi virus (ToLCNDV) the level of miR159 was increased. In the same studies it was also shown that the symptom development may also be due to the upregulation of this miRNA [44].

TYLCV REn interacts with at least two host-encoded proteins, PCNA and the RBR that play an important role in altering the cell cycle [45]. A major function of RBR proteins is to control the expression of many genes required for cell cycle progression, by regulating the activity of E2F transcription factors [46]. The study here has shown a further way that REn may influence the cell-cycle of the host, by up-regulating miR159. CbLCuV has been shown to alter expression of cell cycle-associated genes, preferentially activating genes expressed during the S and G2 phases as well as inhibiting genes active in G1 and M phases. A limited set of core cell cycle genes associated with cell cycle reentry, late G1, S, and early G2 had increased RNA levels, while core cell cycle genes linked to early G1 and late G2 had reduced transcripts [47].

miR160 is encoded on chromosome 2 in *Arabidopsis *[8] and targets mRNAs coding for auxin response factor (ARF) proteins [9,31]. ARFs are a major class of transcription activators and repressors that facilitate the auxin signal by binding to specific cis-elements in the upstream regions of auxin-inducible genes [48]. The study presented here has shown a basic difference in the infection patterns of OW bipartite and monopartite viruses. ACMV infection resulted in decreased accumulation of miR159, whereas for the OW monopartite virus infection resulted in the increased accumulation of miR160. The major role in these interaction could be of βC1 in case CLCuMV/CLCuMB and TrAP/V2 of TYLCV because the phenotypes produced by V2 of TYLCV and βC1, when expressed from the PVX vector in *N. benthamiana*, produced virus-like symptoms [49,50]. These symptoms suggest that the auxin response has been disturbed. The finding that CLCuMV/CLCuMB, TYLCV and CbLCuV infections of *N. benthamiana *resulted in an increase in the levels of miR160 also suggest a general behavior of begomoviruses infection in reducing the response to auxin in infected plants, although this was not the case for ACMV. This suggestion will need further experimental confirmation. Earlier studies with the *Curtovirus *BCTV showed that infection reduces auxin levels, but the authors were unable to show a correlation between reduced auxin and visible symptoms [51].

The miRNA miR164 negatively regulates several genes that encode NAC-like transcription factors [7,31,52,53]. These genes include CUP-SHAPED COTYLEDON 1 (CUC1) and CUC2, which are expressed in, and are necessary for, the formation of boundaries between meristems and emerging organ primordia [54-56]. Failure to establish organ boundaries leads to severe developmental consequences [57]. Infections of all four begomoviruses resulted in an increased accumulation of miR164, suggesting that the viruses down regulate the NAC-like transcription factors. This effect upon miR164 may be one of the contributing factors to the induction of disease symptoms for these viruses. We have observed that with the exception of CLCuMV, there is a significant increase in the levels of miR164 upon PVX-mediated expression of the TrAP genes of all viruses under study (Amin et al, unpublished data). Therefore, TrAP might be the gene responsible for this interaction of viruses with miR164. It has been shown that the TrAPs of ToLCNDV, *Papaya leaf curl virus *(PaLCuV) and CLCuKoV can counter a HR induced by NSP (ToLCNDV) or V2 (PaLCuV and CLCuKoV [58,59]. Virus up-regulation of miR164 may provide a possible explanation of this phenomenon. A recent study has shown that oxygen responsive elements 1 (ORE1), which is a NAC-like transcription factor, positively regulates aging-induced cell death in *Arabidopsis thaliana *leaves. ORE1 expression is up-regulated concurrently with leaf aging by ethylene insensitive 2 (EIN2) but is negatively regulated by miR164. miR164 expression gradually decreases with aging through negative regulation by EIN2, which leads to the up-regulation of ORE1 expression [60] and thus to the cell death. Up-regulation of miR164 thus will counter the cell death, and thus possibly also HR associated cell death due to NSP and V2.

A uniform pattern of up-regulation was observed with relation to the accumulation of miRNA165/166. miR165 is found on chromosome 1 in *Arabidopsis *and regulates HD-ZIPIII transcription factor genes, PHABULOSA (PHB) and PHAVOLUTA (PHV; [9]). It has been shown in *Arabidopsis *that the establishment of leaf polarity requires the generation and perception of positional information along the radial axis of the plant [61]. The results presented here also showed that in general, begomovirus infection resulted in increased accumulation of miR165/166. The genes which upon inoculation produced virus like symptoms may be involved in this interaction. It has been earlier reports that transgenic *Arabidopsis *expressing the TYLCCNB βC1 exhibited virus-like symptoms. These morphological changes were paralleled by a reduction in miR165/166 levels and an increase in PHB and PHV transcript levels. Two factors, ASYMMETRIC LEAVES 1 (AS1) and ASYMMETRIC LEAVES 2 (AS2), are known to regulate leaf development as an AS1/AS2 complex [62]. βC1 is able to partially complement *as2 *mutation. We also observed significant decrease in the levels of miR165/166 upon PVX-mediated expression of CLCuMB βC1 (Amin et al., unpublished data), CLCuMV/CLCuMB infection led to an increased accumulation.

The results show that begomovirus infection generally increases the accumulation of miR167. It has been shown that miR167 targets ARF 6 and ARF8 [9,63]. ARF proteins regulate embryogenesis, root development and floral organ formation [63-67]. ARF6 and ARF8 regulate flower maturation [68]. Infection of plants with some begomoviruses, as well as constitutive expression of some of their genes in plants results in severe developmental defects. For example, transgenic expression of ACMV AC4 in *Arabidopsis *resulted in stunted plants with severe developmental defects, such as narrow rosette leaves and lack of development of reproductive tissue [33]. Similarly the transgenic expression of βC1 in *N. benthamiana *as well as expression on βC1 from PVX resulted in severely twisted plants [49,69,70]. Thus, the results presented here suggest a possible mechanism for the induction of these virus-like symptoms.

With the exception of CbLCuV, infection of *N. benthamiana *plants with all viruses under study resulted in a slightly increased accumulation in the levels of both miR169 and miR170 which suggests that begomovirus infection does not significantly affect the levels of these miRNAs. It has been shown that miR169 and related miRNAs are strongly dependent on P or N status in *Arabidopsis *and rapeseed (*Brassica napus*) phloem sap, flagging them as candidate systemic signaling molecules [71].

The study presented here has shown that, in general, begomovirus infection (assuming that the four viruses originating from distinct classes of begomoviruses [NW, OW, bipartite, monopartite and betasatellite requiring] are representative) increases the accumulation of miRNAs. This finding is in agreement with an earlier study of RNA viruses from three distinct families (*Tobamoviridae*, *Potyviridae*, and *Potexviridae*) that examined a similar range of miRNAs [34]. However, it is difficult to reconcile the presence for all these viruses (both the begomoviruses and the RNA viruses) of proteins that apparently bind (and presumably inactivate/down-regulate) miRNAs with a system that ultimately leads to miRNA up-regulation.

## Methods

### Virus Infections

Agroinoculable infectious clones of begomovirus isolates CLCuMV-His[PK:Mul] (AJ496461), ACMV-[CM:DO3:98] (DNA-A, AY211885; DNA-B, AF112353), CbLCuV-[US:Flo:96] (DNA-A, U65529; DNA-B, U65530) and TYLCV-Mld[ES:72:97] (AF071228) were used to infect *N. benthamiana *plants. Three leaves per plants were inoculated. Samples were collected at 21 dpi. The whole experiment was done on two independent biological replicates.

### miRNA Analysis

Total RNA was isolated from leaves using TRIzol reagent (Invitrogen, Carlsbad, CA), 1/5 volume of 5× RNA loading dye (95% deionized formamide, 0.025% bromophenol blue, 0.025%Xylene cyanol FF, 5 mM EDTA (pH8), 0.025% formaldehyde; 20 μl of 10 mg/ml ethidium bromide was added per 2 ml of dye) was added to 30 μg of total RNA. After heating that at 65°C for 5 min, sample was placed on ice for 2 min before loading it to TBE gel (15%TBE; 7 M urea). Gel was run in 1× TBE buffer at 150-180 volts for 2 to 2.5 hours. After removing gel from the cassette it was photographed under UV trans-illumination and details of the samples were documented.

RNA was transferred to Hybond N+ (Amersham) by a semi-dry blotting system (Bio-Rad) at 10-12 volts for 45-60 min. The membrane was air dried, UV cross-linked and stored at 4°C between two sheets of wet Whatman filter paper.

Oligonucleotide primers complementary to *Arabidopsis *miRNAs (miR156, miR159, miR160, miR164, miR165, miR166, miR167, miR168, miR169 and miR170) were end-labeled using a DIG olig-labeling kit according to manufacturer instructions (Roche, USA). The sequences of oligo-nucleotides used for end labeling are listed in Table 1. Blot was hybridized with probe at 42°C for 12-16 hours. Washing was done with 2XSSC, 1% SDS and 1× SSC, 0.1%SDS for 30 min each. The blot was developed by using the CDP-Star method according to the manufacturer instruction (Roche) and image was taken on X-ray film (hyper film, Amersham, UK).

**Table 1:** Name and sequence of oligonucletides used for end labeling

miRNA	Sequence
**miR156**	**5'**-TGACAGAAGAGAGTGAGCAC**-3'**
**miR159**	**5'- **TTTGGATTGAAGGGAGCTCTA-**3'**
**miR160**	**5'- **TGCCTGGCTCCCTGTATGCCA-**3'**
**miR164**	**5'-**TGGAGAAGCAGGGCACGTGCA**-3'**
**miR165**	**5'- **TCGGACCAGGCTTCATCCCCC **-3'**
**miR166**	**5'- **TCGGACCAGGCTTCATTCCCC -**3'**
**miR167**	**5'- **TGAAGCTGCCAGCATGATCTA **-3'**
**miR168**	**5'- **TCGCTTGGTGCAGGTCGGGAA **-3'**
**miR169**	**5'- **CAGCCAAGGATGACTTGCCGA **-3'**
**miR170**	**5'- **TGATTGAGCCGTGTCAATATC **-3'**

The intensity of bands was quantified by using software ImageJ. Data from these analyses were used to normalize the intensity of each band, based on rRNA loaded in each well. For virus infection the values for the miRNA species in non-infected *N. benthamiana *plants were set at 1 and other data calculated relative to this value. The data shown in Figure 2 is the average of two independent biological replicates along with standard deviation (SD).

### Experimental design

A group of 15 *N. benthamiana *plants were agro-infiltrated with infectious clones of ACMV, CLCuMV/CLCuMB, CbLCuV and TYLCV in two independent experiments. Potato virus X (PVX) was used as reference for virus infection with an RNA genome. All plants were kept in the same green house for duration of each experiment.

## Competing interests

The authors declare that they have no competing interests.

## Authors' contributions

IA, BP performed the experiments. IA, RWB, SM and CMF conceived the study and wrote the manuscript. All authors read and approved the final manuscript.

## References

[B1] Bartel DP MicroRNAs: Genomics, biogenesis, mechanism, and function Cell 2004 116 281 297 10.1016/S0092-8674(04)00045-5 14744438

[B2] Voinnet O Origin, biogenesis, and activity of plant microRNAs Cell 2009 136 669 687 10.1016/j.cell.2009.01.046 19239888

[B3] Lee RC Feinbaum RL Ambros V The *C. elegans *heterochronic gene lin-4 encodes small RNAs with antisense complementarity to lin-14 Cell 1993 75 843 854 10.1016/0092-8674(93)90529-Y 8252621

[B4] Pasquinelli AE Ruvkun G Control and developmental timing by microRNAs and their targets Annu Rev Cell Dev Biol 2002 18 495 513 10.1146/annurev.cellbio.18.012502.105832 12142272

[B5] Llave C Kasschau KD Rector MA Carrington JC Endogenous and silencing-associated small RNAs in plants Plant Cell 2002 14 1605 1619 10.1105/tpc.003210 12119378 PMC150710

[B6] Mette MF Kanno T Aufsatz W Jakowitsch J van der Winden J Matzke MA Matzke AJM Endogenous viral sequences and their potential contribution to heritable virus resistance in plants EMBO J 2002 21 461 469 10.1093/emboj/21.3.461 11823438 PMC125834

[B7] Park W Li J Song R Messing J Chen X CARPEL FACTORY, a Dicer homolog, and HEN1, a novel protein, act in microRNA metabolism in Arabidopsis thaliana Curr Biol 2002 12 1484 1495 10.1016/S0960-9822(02)01017-5 12225663 PMC5137372

[B8] Reinhart BJ Weinstein EG Rhoades MW Bartel B Bartel DP MicroRNAs in plants Genes Dev 2002 16 1616 1626 10.1101/gad.1004402 12101121 PMC186362

[B9] Rhoades M Reinhart B Lim L Burge C Bartel B Bartel DP Prediction of plant microRNA targets Cell 2002 110 513 520 10.1016/S0092-8674(02)00863-2 12202040

[B10] Llave C Xie Z Kasschau KD Carrington JC Cleavage of Scarecrow-like mRNA targets directed by a class of *Arabidopsis *miRNA Science 2002 297 2053 2056 10.1126/science.1076311 12242443

[B11] Tang G Reinhart BJ Bartel DP Zamore PD A biochemical framework for RNA silencing in plants Genes Dev 2003 17 49 63 10.1101/gad.1048103 12514099 PMC195971

[B12] Baumberger N Baulcombe DC *Arabidopsis *ARGONAUTE1 is an RNA slicer that selectively recruits microRNAs and short interfering RNAs Proc Natl Acad Sci USA 2005 102 11928 11933 10.1073/pnas.0505461102 16081530 PMC1182554

[B13] Qi Y Denli AM Hannon GJ Biochemical specialization within *Arabidopsis *RNA silencing pathways Mol Cell 2005 19 421 428 10.1016/j.molcel.2005.06.014 16061187

[B14] Aukerman MJ Sakai H Regulation of flowering time and floral organ identity by a microRNA and its APETALA2-like target genes Plant Cell 2003 15 2730 2741 10.1105/tpc.016238 14555699 PMC280575

[B15] Schwab R Palatnik JF Riester M Schommer C Schmid M Weigel D Specific effects of microRNAs on the plant transcriptome Dev Cell 2005 8 517 527 10.1016/j.devcel.2005.01.018 15809034

[B16] Arteaga-Vazquez M Caballero-Perez J Vielle-Calzada JP A family of microRNAs present in plants and animals Plant Cell 2006 18 3355 3369 10.1105/tpc.106.044420 17189346 PMC1785418

[B17] Gandikota M Birkenbihl RP Hohmann S Cardon GH Saedler H Huijser P The miRNA156/157 recognition element in the 3' UTR of the *Arabidopsis *SBP box gene SPL3 prevents early flowering by translational inhibition in seedlings Plant J 2007 49 683 693 10.1111/j.1365-313X.2006.02983.x 17217458

[B18] Hanley-Bowdoin L Settlage SB Orozco BM Nagar S Robertson D Geminviruses: models for plant DNA replication, transcription, and cell cycle regulation Crit Rev Plant Sci 1999 18 71 106 10.1016/S0735-2689(99)00383-4 10821479

[B19] Stanley J Bisaro DM Briddon RW Brown JK Fauquet CM Harrison BD Rybicki EP Stenger DC Fauquet CM, Mayo MA, Maniloff J, Desselberger U, Ball LA Geminiviridae Virus Taxonomy, VIIIth Report of the ICTV 2005 London: Elsevier/Academic Press 301 326

[B20] Varma A Malathi VG Emerging geminivirus problems: A serious threat to crop production Ann Appl Biol 2003 142 145 164 10.1111/j.1744-7348.2003.tb00240.x

[B21] Mansoor S Briddon RW Zafar Y Stanley J Geminivirus disease complexes: an emerging threat Trends Plant Sci 2003 8 128 134 10.1016/S1360-1385(03)00007-4 12663223

[B22] Nawaz-ul-Rehman MS Fauquet CM Evolution of geminiviruses and their satellites FEBS Lett 2009 583 1825 1832 10.1016/j.febslet.2009.05.045 19497325

[B23] Dry I Krake LR Rigden JE Rezaian MA A novel subviral agent associated with a geminivirus: the first report of a DNA satellite Proc Natl Acad Sci, USA 1997 94 7088-7093 10.1073/pnas.94.13.7088 PMC21289 9192696

[B24] Zaitlin M Hull R Plant Virus-Host Interaction Annu Rev Plant Physio 1987 38 291 315 10.1146/annurev.pp.38.060187.001451

[B25] Finnegan EJ Matzke MA The small RNA world J Cell Sci 2003 116 4689 4693 10.1242/jcs.00838 14600255

[B26] Voinnet O Non-cell autonomous RNA silencing: insights from viral infections FEBS Lett 2005 579 5858 10.1016/j.febslet.2005.09.039 16242131

[B27] Dunoyer P Voinnet O The complex interplay between plant viruses and host RNA-silencing pathways Curr Opin Plant Biol 2005 8 415 10.1016/j.pbi.2005.05.012 15939663

[B28] Pfeffer S Voinnet O Viruses, microRNAs and cancer Oncogene 2006 25 6211 6219 10.1038/sj.onc.1209915 17028601

[B29] Roth BM Pruss GJ Vance VB Plant viral suppressors of RNA silencing Virus Res 2004 102 97 108 10.1016/j.virusres.2004.01.020 15068885

[B30] Chapman EJ Prokhnevsky AI Gopinath K Dolja VV Carrington JC Viral RNA silencing suppressors inhibit the microRNA pathway at an intermediate step Genes Dev 2004 18 1179 1186 10.1101/gad.1201204 15131083 PMC415642

[B31] Kasschau KD Xie Z Allen E Llave C Chapman EJ Krizan KA Carrington JC P1/HC-Pro, a viral suppressor of RNA silencing, interferes with *Arabidopsis *development and miRNA function Dev Cell 2003 4 205 217 10.1016/S1534-5807(03)00025-X 12586064

[B32] Vanitharani R Chellappan P Pita JS Fauquet CM Differential roles of AC2 and AC4 of cassava geminiviruses in mediating synergism and suppression of posttranscriptional gene silencing J Virol 2004 78 9487 9498 10.1128/JVI.78.17.9487-9498.2004 15308741 PMC506916

[B33] Chellappan P Vanitharani R Fauquet CM MicroRNA-binding viral protein interferes with *Arabidopsis *development Proc Natl Acad Sci USA 2005 102 10381 10386 10.1073/pnas.0504439102 16006510 PMC1177406

[B34] Bazzini AA Hopp HE Beachy RN Asurmendi S Infection and coaccumulation of tobacco mosaic virus proteins alter microRNA levels, correlating with symptom and plant development Proc Natl Acad Sci USA 2007 104 12157 12162 10.1073/pnas.0705114104 17615233 PMC1924585

[B35] Baulcombe D RNA silencing Trends Biochem Sci 2005 30 290 293 10.1016/j.tibs.2005.04.012 15950871

[B36] Voinnet O Induction and suppression of RNA silencing: insights from viral infections Nature Genet 2005 6 206 221 10.1038/nrg1555 15703763

[B37] Voinnet O RNA silencing as a plant immune system against viruses Trends Genet 2001 17 449 459 10.1016/S0168-9525(01)02367-8 11485817

[B38] Zrachya A Glick E Levy Y Arazi T Citovsky V Gafni Y Suppressor of RNA silencing encoded by *Tomato yellow leaf curl virus*-Israel Virology 2007 358 159 165 10.1016/j.virol.2006.08.016 16979684

[B39] Riechmann JL Heard J Martin G Reuber L Jiang C Keddie J Adam L Pineda O Ratcliffe OJ Samaha RR *Arabidopsis *transcription factors: genome-wide comparative analysis among eukaryotes Science 2000 290 2105 2110 10.1126/science.290.5499.2105 11118137

[B40] Wu G Poethig RS Temporal regulation of shoot development in *Arabidopsis *thaliana by miR156 and its target SPL3 Development 2006 133 3539 3547 10.1242/dev.02521 16914499 PMC1610107

[B41] Mette MF van der Winden J Matzke M Matzke AJ Short RNAs can identify new candidate transposable element families in *Arabidopsis* Plant Physiol 2002 130 6 9 10.1104/pp.007047 12226481 PMC1540252

[B42] Parcy F Bomblies K Weigel D Interaction of LEAFY, AGAMOUS and TERMINAL FLOWER1 in maintaining floral meristem identity in Arabidopsis Development 2002 129 2519 2527 11973282 10.1242/dev.129.10.2519

[B43] Blazquez MA Soowal LN Lee I Weigel D LEAFY expression and flower initiation in *Arabidopsis* Development 1997 124 3835 3844 9367439 10.1242/dev.124.19.3835

[B44] Naqvi AR Haq QM Mukherjee SK MicroRNA profiling of *Tomato leaf curl New Delhi virus *(ToLCNDV) infected tomato leaves indicates that deregulation of mir159/319 and mir172 might be linked with leaf curl disease Virol J 2010 7 281 10.1186/1743-422X-7-281 20973960 PMC2972279

[B45] Settlage SB See RG Hanley-Bowdoin L Geminivirus C3 protein: replication enhancement and protein interactions J Virol 2005 79 9885 9895 10.1128/JVI.79.15.9885-9895.2005 16014949 PMC1181577

[B46] Sabelli PA Larkins BA Regulation and function of retinoblastoma-related plant genes Plant Sci 2009 177 540 548 10.1016/j.plantsci.2009.09.012

[B47] Ascencio-Ibanez JT Sozzani R Lee TJ Chu TM Wolfinger RD Cella R Hanley-Bowdoin L Global analysis of *Arabidopsis *gene expression uncovers a complex array of changes impacting pathogen response and cell cycle during geminivirus infection Plant Physiol 2008 148 436 454 10.1104/pp.108.121038 18650403 PMC2528102

[B48] Guilfoyle TJ Hagen G Auxin response factors J Plant Growth Regul 2001 10 281 291 10.1007/s003440010026

[B49] Qazi J Amin I Mansoor S Iqbal MJ Briddon RW Contribution of the satellite encoded gene βC1 to cotton leaf curl disease symptoms Virus Res 2007 128 135 139 10.1016/j.virusres.2007.04.002 17482706

[B50] Selth LA Randles JW Rezaian MA Host responses to transient expression of individual genes encoded by *Tomato leaf curl virus* Mol Plant Microbe In 2004 17 27 33 10.1094/MPMI.2004.17.1.27 14714865

[B51] Smith SH McCall SR Harris JH Alterations in the auxin levels of resistant and susceptible hosts induced by curly top virus Phytopathology 1968 58 1669 1670

[B52] Baker CC Sieber P Wellmer F Meyerowitz EM The early extra petals1 mutant uncovers a role for microRNA miR164c in regulating petal number in Arabidopsis Curr Biol 2005 15 303 315 10.1016/j.cub.2005.02.017 15723790

[B53] Guo HS Xie Q Fei JF Chua NH MicroRNA directs mRNA cleavage of the transcription factor NAC1 to downregulate auxin signals for *Arabidopsis *lateral root development Plant Cell 2005 17 1376 1386 10.1105/tpc.105.030841 15829603 PMC1091761

[B54] Aida M Ishida T Tasaka M Shoot apical meristem and cotyledon formation during *Arabidopsis *embryogenesis: interaction among the CUP-SHAPED COTYLEDON and SHOOT MERISTEMLESS genes Development 1999 126 1563 1570 10079219 10.1242/dev.126.8.1563

[B55] Heisler MG Ohno C Das P Sieber P Reddy GV Long JA Meyerowitz EM Patterns of auxin transport and gene expression during primordium development revealed by live imaging of the *Arabidopsis *inflorescence meristem Curr Biol 2005 15 1899 1911 10.1016/j.cub.2005.09.052 16271866

[B56] Takada S Hibara K Ishida T Tasaka M The CUP-SHAPED COTYLEDON1 gene of *Arabidopsis *regulates shoot apical meristem formation Development 2001 128 1127 1135 11245578 10.1242/dev.128.7.1127

[B57] Aida M Ishida T Fukaki H Fujisawa H Tasaka M Genes involved in organ separation in *Arabidopsis*: an analysis of the cup-shaped cotyledon mutant Plant Cell 1997 9 841 857 10.1105/tpc.9.6.841 9212461 PMC156962

[B58] Hussain M Mansoor S Iram S Zafar Y Briddon RW The hypersensitive response to *Tomato leaf curl New Delhi virus *nuclear shuttle protein is inhibited by transcriptional activator protein Mol Plant Microbe In 2007 20 1581 1588 10.1094/MPMI-20-12-1581 17990965

[B59] Mubin M Amin I Amrao L Briddon RW Mansoor S The hypersensitive response induced by the V2 protein of a monopartite begomovirus is countered by the C2 protein Mol Plant Pathol 2010 11 245 254 10.1111/j.1364-3703.2009.00601.x 20447273 PMC6640282

[B60] Kim JH Woo HR Kim J Lim PO Lee IC Choi SH Hwang D Nam HG Trifurcate feed-forward regulation of age-dependent cell death involving miR164 in Arabidopsis Science 2009 323 1053 1057 10.1126/science.1166386 19229035

[B61] Mallory AC Reinhart BJ Jones-Rhoades MW Tang G Zamore PD Barton MK Bartel DP MicroRNA control of PHABULOSA in leaf development: importance of pairing to the microRNA 5' region EMBO J 2004 23 3356 3364 10.1038/sj.emboj.7600340 15282547 PMC514513

[B62] Xu L Yang L Pi L Liu Q Ling Q Wang H Poethig RS Huang H Genetic interaction between the AS1-AS2 and RDR6-SGS3-AGO7 pathways for leaf morphogenesis Plant Cell Physiol 2006 47 853 863 10.1093/pcp/pcj057 16699177

[B63] Wang JW Wang LJ Mao YB Cai WJ Xue HW Chen XY Control of root cap formation by microRNA-targeted auxin response factors in *Arabidopsis* Plant Cell 2005 17 2204 2216 10.1105/tpc.105.033076 16006581 PMC1182483

[B64] Hardtke CS Berleth T The *Arabidopsis *gene MONOPTEROS encodes a transcription factor mediating embryo axis formation and vascular development EMBO J 1998 17 1405 1411 10.1093/emboj/17.5.1405 9482737 PMC1170488

[B65] Hardtke CS Ckurshumova W Vidaurre DP Singh SA Stamatiou G Tiwari SB Hagen G Guilfoyle TJ Berleth T Overlapping and non-redundant functions of the *Arabidopsis *auxin response factors MONOPTEROS and NONPHOTOTROPIC HYPOCOTYL 4 Development 2004 131 1089 1100 10.1242/dev.00925 14973283

[B66] Mallory AC Bartel DP Bartel B MicroRNA-directed regulation of *Arabidopsis *AUXIN RESPONSE FACTOR17 is essential for proper development and modulates expression of early auxin response genes Plant Cell 2005 17 1360 1375 10.1105/tpc.105.031716 15829600 PMC1091760

[B67] Sessions A Nemhauser JL McColl A Roe JL Feldmann KA Zambryski PC ETTIN patterns the *Arabidopsis *floral meristem and reproductive organs Development 1997 124 4481 4491 9409666 10.1242/dev.124.22.4481

[B68] Nagpal P Ellis CM Weber H Ploense SE Barkawi LS Guilfoyle TJ Hagen G Alonso JM Cohen JD Farmer EE Ecker JR Reed JW Auxin response factors ARF6 and ARF8 promote jasmonic acid production and flower maturation Development 2005 132 4107 4118 10.1242/dev.01955 16107481

[B69] Saeed M Behjatania SAA Mansoor S Zafar Y Hasnain S Rezaian MA A Single complementrary-sense transcript of a geminiviral DNA β satellite is determinant of pathogenicity Mol Plant Microbe In 2005 18 7 14 10.1094/MPMI-18-0007 15672813

[B70] Saunders K Norman A Gucciardo S Stanley J The DNA β satellite component associated with ageratum yellow vein disease encodes an essential pathogenicity protein (βC1) Virology 2004 324 37 47 10.1016/j.virol.2004.03.018 15183051

[B71] Pant BD Musialak-Lange M Nuc P May P Buhtz A Kehr J Walther D Scheible WR Identification of nutrient-responsive *Arabidopsis *and rapeseed microRNAs by comprehensive real-time polymerase chain reaction profiling and small RNA sequencing Plant Physiol 2009 150 1541 1555 10.1104/pp.109.139139 19465578 PMC2705054

